# Alkali Metal Doping for Improved CH_3_NH_3_PbI_3_ Perovskite Solar Cells

**DOI:** 10.1002/advs.201700131

**Published:** 2017-12-21

**Authors:** Wangen Zhao, Zhun Yao, Fengyang Yu, Dong Yang, Shengzhong (Frank) Liu

**Affiliations:** ^1^ Key Laboratory for Applied Surface and Colloid Chemistry National Ministry of Education Shaanxi Engineering Lab for Advanced Energy Technology School of Materials Science and Engineering Shaanxi Normal University Xi'an 710062 China; ^2^ Dalian National Laboratory for Clean Energy iChEM (Collaborative Innovation Center of Chemistry for Energy Materials) Dalian Institute of Chemical Physics Chinese Academy of Sciences Dalian 116023 China

**Keywords:** alkali metals, doping, perovskites, planar construction, passivation

## Abstract

Organic–inorganic hybrid halide perovskites are proven to be a promising semiconductor material as the absorber layer of solar cells. However, the perovskite films always suffer from nonuniform coverage or high trap state density due to the polycrystalline characteristics, which degrade the photoelectric properties of thin films. Herein, the alkali metal ions which are stable against oxidation and reduction are used in the perovskite precursor solution to induce the process of crystallization and nucleation, then affect the properties of the perovskite film. It is found that the addition of the alkali metal ions clearly improves the quality of perovskite film: enlarges the grain sizes, reduces the defect state density, passivates the grain boundaries, increases the built‐in potential (*V*_bi_), resulting to the enhancement in the power conversion efficiency of perovskite thin film solar cell.

## 1 Introduction

Organic–inorganic hybrid halide perovskites commonly adopting the ABX_3_ structure: where A is a monovalent organic or inorganic cation, e.g., methylammonium (MA^+^), formamidinium (FA^+^), Cs^+^ etc.; B is a divalent metal ion (Pb^2+^, Sn^2+^, Ge^2+^ etc.) and X is a monovalent anion (Cl^−^, Br^−^, I^−^, SCN^−^ etc.) have attracted great attention owing to their superior photoelectronic properties.^1^, ^2^, ^3^, ^4^, ^5^ As a type of star material in the photovoltaic field, the performance of perovskite‐based thin film solar cells have gone through tremendous advance, almost caught up with or even gone beyond the efficiency of crystalline silicon, CIGSe (copper–indium–gallium diselenide) and CdTe solar cells only in a few years.^1^, ^6^, ^7^, ^8^, ^9^, ^10^ And the high efficiency perovskite solar cell depends on the high‐quality absorbing layer governed by its deposition process including nucleation and thin film growth. However, some undesirable properties, such as the phase transition, instability to humidity, UV (ultraviolet), and heat are often accompanied due to perovskite absorber material own properties.^11^, ^12^, ^13^ In addition, the perovskite films with the polycrystalline nature which crack in the periodic crystal structure lead to the formation of a large number of grain boundaries (GBs). These GBs in the perovskite layer are proven to be recombination centers, which has negative impact on device performance.^14^, ^15^ So, it is desired to prepare larger crystallites with reduced GBs for higher performance. Therefore, further treatment for defect passivation or reduction of GBs is critically vital.^16^, ^17^, ^18^

In an effort to improve the quality of perovskite absorber layer, variations of techniques have been developed, which can be divided into two major types (the optimization of the precursor solution or the perovskite film), by and large. In this regard, combination of solvent and dopants have been tested, such as: the introduction of different source of metal or the solvent, the utilization of single or mixed solvent,^19^, ^20^ a variety of lead salt precursors,^21^, ^22^ as well as so‐called engineering step^23^ or the exotic additive: ionic liquid,^24^ Lewis base^25^, ^26^, ^27^ or other organic and inorganic additives (MACl,^28^ Guanidinium,^29^ water,^30^, ^31^ H_3_PO_2_,^32^, ^33^ 1,8‐diiodooctane (DIO),^34^ 1‐chloronaphthalene (CN),^35^ hydroiodic acid (HI),^36^, ^37^ aluminum acetylacetonate (Al‐acac_3_),^38^ LiI,^39^ Na^+^ 40 etc.), which induce the nucleation and crystallization process of perovskite thin films. Another method is based on solvent vapor fumigation‐induced technology using the pyridine,^41^ water vapor,^42^, ^43^ DMF (anhydrous *N*,*N*‐dimethylformamide)^43^, ^44^, ^45^ and so on, which provide some successful cases by adjusting the recrystallization process of the perovskite film. Both above techniques have been demonstrated to improve film quality directly or indirectly, allowing for the formation of much smoother films with higher surface coverage. However, the effect of these additives on the absorbing layer and device performance is not yet to be investigated in‐depth or not well‐controlled.

In all kinds of dopants, the alkali metal cations (Na^+^, K^+^) have been chosen to be the positive additive owing to their stability or resistance against oxidation and reduction.^46^ Herein, we found that alkali‐metal‐cation additives effectively improved the perovskite film with fewer GBs and traps states, enhanced the built‐in potential, attributing to higher power conversion efficiency (PCE) via assembling device. Meanwhile some auxiliary tests were conducted to reveal the distribution of alkali‐metal‐cation to refer to the possible effect mechanism.

## 2 Results and Discussion

**Figure 1:**
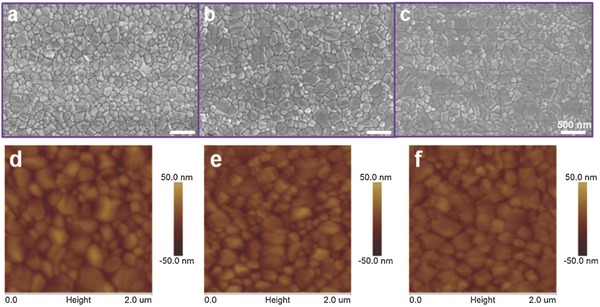
The top‐view FE‐SEM (field emission scanning electron microscopy) images and atom force topology of perovskite film a,d) without, or with b,e) NaI, and c,f) KI doping.

First, to check the quality of perovskite absorber layer, morphological characterization based on field‐emission scanning electron microscopy (FE‐SEM) was carried out to determine the shape and coverage of the MAPbI_3_ grains and thin films prepared in the presence of additives and the control sample (without any additive). It was found that all of the perovskite films exhibited high coverage, which are displayed in **Figure** **1**a–c. Comparative SEM analysis brought out the variations in film evenness and grain size. For a clear comparison, Figure S1 (Supporting Information) provides statistical grain size distribution based on the top‐view images in Figure 1. It is exhibited that without additive, average grain size is ≈140 nm, and the average grain sizes increase to ≈220, 230 nm when Na^+^ and K^+^ doping were used, respectively. Meanwhile, lower surface roughness of perovskite film with alkali metal cation doped are observed compared with the control sample by AFM (atomic force microscope) in Figure 1d–f, with the root‐mean‐square of *R*_q_ = 8.99 (Na doped), 7.94 (K doped), 9.91 (control sample) nm. The perovskite films also appear to be entirely covered based on AFM, being consistent with the FE‐SEM results. Synthesizing above consequences, we found that the additives apparently help to improve the perovskite growth into much large crystallite grains and smoother films.

**Figure 2:**
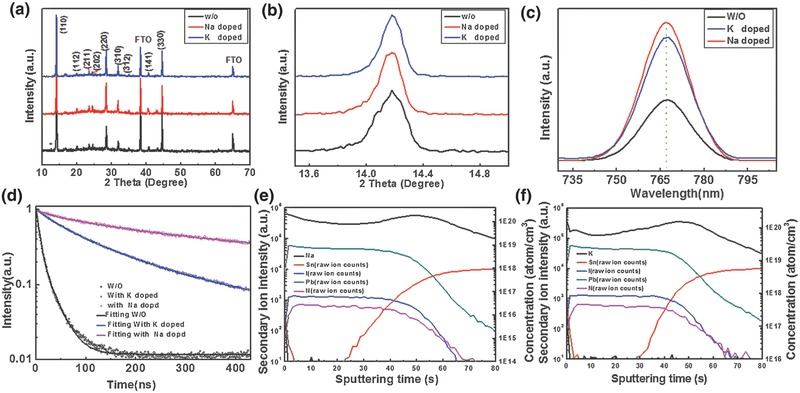
a) The XRD (X‐ray diffraction) patterns, b) the enlarged comparison of (110) diffraction peak, c) the steady‐state photoluminescence (PL) spectra, and d) time‐resolved photoluminescence (TR‐PL) spectroscopy based on the glass/perovskite structure with Na^+^, K^+^ doped and the control sample without doping. e,f) The element distribution of Na^+^, K^+^ doped perovskite absorber layer measured by SIMS (secondary ion mass spectrometer) (note that the contents of Na and K were quantified by the Si standard while the other element contents were recorded as the raw ion counts).

The X‐ray diffraction (XRD) is a powerful tool in resolving crystallization details. **Figure** **2**a gives the XRD of the three samples in demonstrating the typical patterns attributed to the tetragonal MAPbI_3_ phase irrespective of doping or not. There is also no additional impurity phases found in the doped samples. However, a tiny peak remarked with * was observed belonging to PbI_2_ located at 12.6^ο^ in the sample without doping. In addition, all the films show the strongest diffraction peak at ≈14^o^, which are attributed to the black perovskite phase oriented along (110) direction. The enlarged (110) diffraction peak of the XRD results with significantly narrower width in Figure 2b also revealed that larger grain sizes were achieved by addition of monovalent alkali metal cations based on Scherrer equation.

The steady‐state photoluminescence (PL) is an effective way to detect the trap states in the perovskite absorber layer. In this paper, the signal responses were recorded based on the glass/perovskite structure. It was found that there is indeed an obvious increase in intensity with the addition of Na^+^ and K^+^ (Figure 2c). Without the doping, only minimum PL intensity is detected. It is generally believed that the higher PL intensity, the fewer traps or defects. The differences in PL intensity can be assigned to the passivation affection of monovalent alkali metal cations on the grain boundaries of perovskite film, resulting in reducing trapped states. In other words, the addition of Na^+^ and K^+^ gives a better quality material with fewer traps. Careful examinations show that all PL peaks of the perovskite films, without or with the alkali metal cation doping are centered around 767 nm without any detectable shifts. To further experimentally elucidate the relationship between device performance and carrier recombination, time‐resolved photoluminescence (TR‐PL) spectroscopy is performed on the perovskite films in Figure 2d. Because no ETL (electron transport layer) or HTL (hole transport layer) is used, carrier recombination is considered to represent intralayer carrier transport process, which is expected to be strongly dependent on the impurities level at GBs. The perovskite films with lower level of impurities at GBs would show longer carrier lifetimes and slower intralayer recombination rates. By fitting the TR‐PL, the control sample gives an average carrier lifetime of 16.58 ns, while the Na^+^ and K^+^ doped perovskite films increase the average lifetimes to 259.72 and 135.16 ns, as listed in **Table** **1**. These measurements uncover that alkali metal cations are capable of substantially retarding the recombination within the perovskite due to longer carrier lifetime. The longer carrier lifetime corresponds to the higher steady PL, also illustrating the fewer trap states in the doped perovskite films.

**Table 1:** The parameters of carrier lifetime by fitting the TR‐PL spectroscopy based on glass/perovskite structure

Samples	τ_ave_ [ns]	τ_1_ [ns]	Amplitude of τ_1_	τ_2_ [ns]	Amplitude of τ_2_
W/O	16.581	23.433	0.285	6.1403	0.715
Na	259.721	34.217	0.157	265.142	0.843
K	135.156	146.782	0.688	39.375	0.312

Secondary‐ion mass spectrometry (SIMS) depth profiles were recorded in Figure 2e,f to investigate the element distributions of perovskite films with Na^+^ and K^+^ doping. It exhibited that the SIMS depth profiling of N, Pb, I distributions have relative similar stable trend while the depth profiling of Na^+^ or K^+^ have an obvious fluctuation. The ups and downs of the alkali metal cations are attributed to their accumulation in the grain boundaries and the material interface. The accumulation of Na or K may result from their passivation effect at the grain boundaries because there are more defect states.^47^

**Figure 3:**
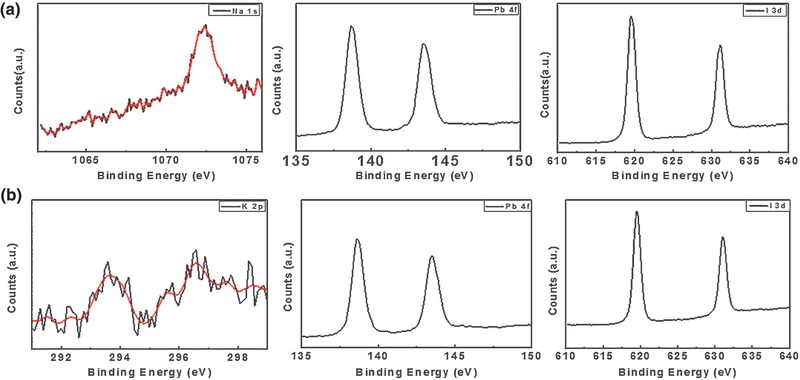
The surface‐sensitive XPS (X‐ray photoelectron spectroscopy) of a) Na^+^ and b) K^+^ doped perovskite film (the measurements were conducted without etching treatment).

Surface‐sensitive X‐ray photoelectron spectroscopy (XPS) measurements were conducted in **Figure** **3** to further verify the existence of monovalent alkali metal cation and valence state of elements consisted in the perovskite film. Both of the perovskite films with Na^+^ and K^+^ addition show two symmetric peaks at ≈143 and 138 eV, which are assigned to the Pb 4f_5/2_ and Pb 4f_7/2_, respectively. These peaks are associated with Pb^2+^ in the perovskite. It was noteworthy to point out that another pair of peaks with lower binding energies (141.6 and 136.8 eV) with the signature of Pb^0^ were detected when the perovskite films were etched by argon ion seen in Figure S3 (Supporting Information).^48^, ^49^ The peaks of Na 1s and K 2p were also observed, confirm the existence of Na^+^ and K^+^. In short, the evident signals from K and Na confirmed that the samples are effectively doped. In addition, the oxygen spectroscopy was only detected before the Ar^+^ etching in Figure S3 (Supporting Information). However, the peak of oxygen would disappear when the surface of perovskite films were treated with Ar^+^ etching, confirming that the O is from the surface oxidation due to the instability of perovskite films.

**Figure 4:**
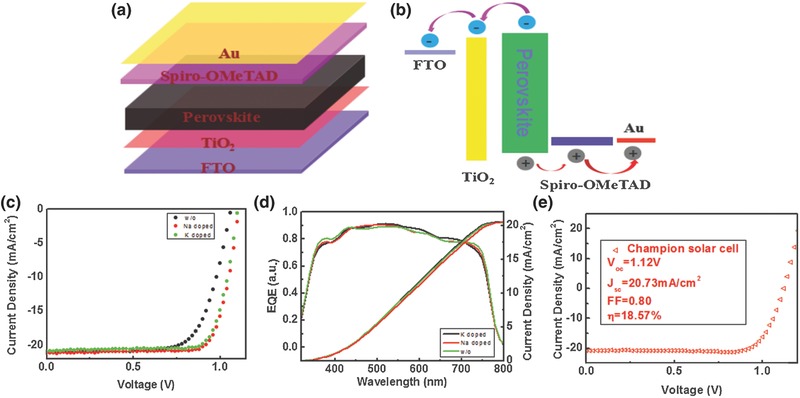
a) The schematic architecture of the perovskite solar cells (PSCs). b) Energy‐level diagram of the PSCs, exhibiting the collecting process of photogenerated carriers. c) The current density–voltage characteristics and d) external quantum efficiency (EQE) spectra of the perovskite device, with Na^+^ or K^+^ doping compared to control sample without doping. e) The *J–V* curve of champion perovskite solar cell with 1.0 mol% Na^+^ doping measured at the AM 1.5G solar spectra of 100 mW cm^−2^ at a reverse scan.

**Figure** **4**a illustrates the cell architecture designed with a traditional structure, wherein the dense TiO_2_ film is used as the ETL, the 2,2′,7,7′‐tetrakis (*N*,*N*‐di‐pmethoxyphenylamine)‐9,9′‐spirobifluorene (spiro‐OMeTAD) as the HTL, and the gold as the top anode. The perovskite film was deposited on the fluorine‐doped tin oxide (FTO) transparent electrode as the absorbing layer by one‐step solvent engineering process. In this typical device structure, suited energy‐level layer was designed to promote the extraction of the electron or hole. Figure 4b simulated the energy‐level diagram of the device, which reveals the electron transport routes. The *J–V* curves of completed perovskite photovoltaic device with the absorber layer doped by alkali metal cation (Na^+^ and K^+^) and the control sample are displayed in Figure 4c with key photovoltaic parameters listed in **Table** **2**. It was observed that both Na^+^ and K^+^ have a positive effect on the performance of perovskite device. Obviously, the PCE of the solar cell is improved from 15.56% to 18.16% (with 1% Na^+^ doped) with an enhancement ratio of ≈17%, attributing to the significant improvement in FF (fill factor) and *V*_oc_ (open‐circuit voltage) due to some advantageous changes in the morphology of the photoactive materials induced by the monovalent cation additive. It shows that FF of both doped cells is improved from 0.70 to 0.78, an increase of 11.4%; while *V*_oc_ is improved from 1.06 to 1.10 V, an increase of 3.8%. This is because that the addition of alkali metal cations passivates the surface and retards the electron–hole recombination resulting in an improvement in *V*_oc_. The enhancement of FF result from the increasing shunt resistance and reducing series resistance due to higher quality absorber layer with reducing the shunt paths and recombination chance for electrons and holes. The integrated current density based on the EQE spectra (Figure 4d) matched well with the *J*_sc_ from *J–V* curve, by and large. Figure 4e also gives the champion efficiency of perovskite solar cell with 1% Na^+^ doing, with a *J*_sc_ = 20.73 mA cm^−2^, FF = 0.80, *V*_oc_ = 1.12 V, η = 18.57%.

**Table 2:** The key photovoltaic parameters of the perovskite solar cell with Na^+^, K^+^ doped, and pristine control perovskite device. The active area of each PSCs were defined by a metal mask as 0.09 cm^2^

	FF	*V*_oc_ [V]	*J*_sc_ [mA cm^−2^]	η [%]
Control	0.70	1.06	20.97	15.56
Na doped	0.78	1.10	21.16	18.16
K doped	0.78	1.10	20.88	17.81

**Figure 5:**
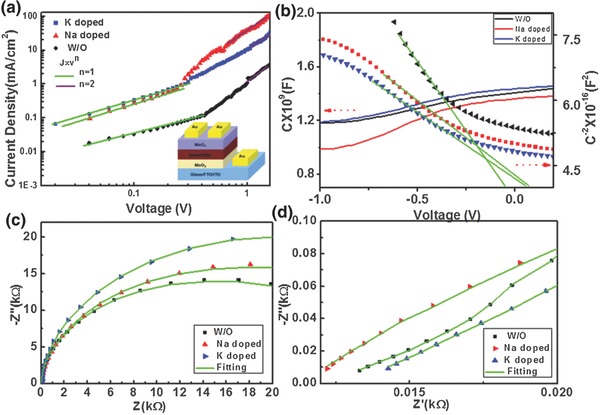
a) Dark *J–V* curves of the hole‐only device revealing *V*_TFL_ (the trap‐filled limit voltage) kink point behavior. The lower‐right inset illustrates the configuration of the hole‐only device. b) *C–V*, 1/*C* ^2^ *–V* curves and c) the Nyquist plot of perovskite solar cells without and with different alkali metals doping. d) The EIS (electrochemical impedance spectroscopy) zoomed in to the high‐frequency region.

The dark current–voltage characteristics of the hole‐only devices were used to evaluate the trap state density of the perovskite film. It was exhibited that the perovskite thin film with addition of monovalent alkali metal cation has a relative low *V*_TFL_ compared with the pristine control sample in **Figure** **5**a. The trap state density of the perovskite film can be calculated by the following equation^50^

(1) $$n_{t}=\frac{2V_{TFL}\;\varepsilon \varepsilon_{0}}{eL^{2}}$$

where *V*_TFL_ is the trap‐filled limit voltage, *L* is the thickness of the film, ε_0_ is the vacuum permittivity, *e* is the electron charge, and ε is the dielectric constant. So, lower *V*_TEF_ was connected with lower concentration of trap states. While the control sample without doping shows a *V*_TFL_ as high as ≈0.48 V, they are effectively reduced by Na and K doping to ≈0.28 and ≈0.3 V, respectively. Therefore, it was conducted that the addition of alkali metal cations have reduced the trap states of the perovskite film. Figure 5b shows the *C*–*V* and 1/*C* ^2^–*V* curves of the PSCs for determining the effective hole carrier concentration of the perovskite absorber layer. Using the Mott–Schottky method, the net carrier concentration can be evaluated based on the Equation (2), 51

(2) $$N_{c}(W)=2\;/\;qK_{s}\varepsilon_{0}A^{2}\;\;\left[d(1\;/\;C^{2})\;/\;dV\right]$$

where *N*_c_(*W*) is the net carrier concentration, *q* is the electron charge, *K_s_* is the semiconductor dielectric constant, ε_0_ is the permittivity of free space, *A* is the area of the solar cell, *C* is the capacitance, and *V* is the applied voltage. It demonstrates that the alkali metal doped perovskite devices have changed carrier concentration based on above equation, and they also have led to a higher built‐in voltage (the point of intersection by epitaxy the curve of 1/*C^2^*–*V* when it is equal to zero). The higher built‐in voltage is favorable for the improvement of open‐circuit voltage (*V*_oc_), which also accounts for the increase of *V*_oc_ for alkali metal cation doped perovskite device as shown in the measurements.

The Nyquist plot and tested AC response equivalent circuit of the perovskite solar cell are displayed in Figures 5c and Figure S5 (Supporting Information). The impedance spectra were recorded in dark by varying the biases at 1.0 V. The first semicircle in high‐frequency region (commonly not clear due to relatively small value) is attributed to charge transfer at the interface of TiO_2_/perovskite and perovskite/spiro‐OMeTAD. The second semicircle (in intermediate and low‐frequency region) is related to the recombination phenomena at the interface of CH_3_NH_3_PbI_3_ and TiO_2_ or hole transport material (HTM) layer. The relative *R* and *C* values are obtained by fitting the spectra with a Zview program. Specific fitting parameters are shown in Table S1 in the Supporting Information. It was seen that the addition of Na^+^ has reduced the *R*_s_ (11.41 vs 13.17 Ω) and increased the *R*_rec_ (14519 vs 10456 Ω) compared to the control sample, which complementally explain the change of main performance parameters of perovskite photovoltaic device.

## 3 Conclusions

An effective additive method has been developed based on one‐step, solution crystallization process to prepare the perovskite film. It has been demonstrated that the addition of alkali metal cations in the perovskite precursors significantly improve the grain size, and reduce the trap states, which is vital for achieving high‐efficiency polycrystalline thin film solar cells. In addition, we observed enhanced luminescent intensity, lengthened carrier lifetime by alkali metal cations doping, which help to push the performance of the planar perovskite solar cells to a higher level. The approach proposed in this work, which is simple and facile yet very effective, allows versatility with widespread applicability since the additive is readily applicable to other bulk‐heterojunction systems with great adaptability, thus facilitating the practical application of photovoltaic technologies.

## 4 Experimental Section

*Chemicals*: PbI_2_, lithium bis (trifluoromethanesulfonyl)imide (Li‐TFSI), 4‐tert‐butyl pyridine (tBP), chlorobenzene were obtained from Sigma‐Aldrich. Spiro‐OMeTAD was purchased from Shenzhen Feiming Technology Co. γ‐butyrolactone, dimethylsulfoxide, HI (57 wt% in water), methylamine (CH_3_NH_2,_ 40 wt% in aqueous solution), diethyl ether, sodium iodide (NaI), potassium iodide (KI) were bought from Aladdin Inc. All the chemicals were used without further purification.

*The Synthesis of CH_3_NH_3_I*: The CH_3_NH_3_I was synthesized using the method described by reference elsewhere.^50^ 15.0 mL of HI aqueous solution was reacted with 13.5 mL methylamine (CH_3_NH_2_) aqueous solution at 0 °C for 2 h with constant stirring under a nitrogen atmosphere. Methylammonium iodide was crystallized through removing the solvent by a rotary evaporator. The generated white powder was achieved by washing with diethyl ether three times and drying under vacuum overnight.

*Preparation of TiO_2_ Dense Layer*: TiO_2_ was deposited on a glass (25 × 25 mm) coated by FTO. The cleaning process of FTO glasses were conducted based on a standard process with washing‐up liquid/acetone/ethanol for 30 min each. Then, the clean FTO glasses were treated under ultraviolet and ozone for about 8 min. The treated FTO was flat out in a Petri dish filled with 40 × 10^−3^ m TiCl_4_ aqueous solution for 60 min at about 70 °C. Finally, the FTO substrates coated with TiO_2_ were washed with distilled water and ethanol, followed by drying with nitrogen.

*Deposition of the Absorbing Layer and the Fabrication of Photovoltaic Device*: The precursor solution was prepared as following: 159 mg CH_3_NH_3_I and 462 mg PbI_2_ were dissolved in 1 mL mixture of γ‐butyrolactone and dimethylsulphoxide (7:3, v/v), and then stirred at room temperature for several hours to attain clear light yellow solution. Then, the prepared solutions were filtered by using a 0.22 µm pore polyvinylidene fluoride syringe filter before spin‐coating. The perovskite thin film was spin‐coated onto the FTO glass substrates by a consecutive two‐step spin‐coating process at 1000 and 4000 rpm for 10 and 40 s, respectively. It was noted that 500 µL chlorobenzene was dripped as antisolvent 15 s after the second stage to obtain a light‐brown smooth perovskite thin film. Then the CH_3_NH_3_PbI_3_ as‐prepared thin film was heated at 100 °C for 10 min on prepared heat plate to convert to a dark‐brown film. After cooling of the perovskite thin film, the spiro‐OMeTAD solution with a concentration of 90 mg mL^−1^ was spin‐coated onto the perovskite films at 5000 rpm (revolutions per minute) for 30 s. It was noted that the 22 µL Li‐TFSI solution (520 mg mL^−1^ in acetonitrile) and 36 µL tBP were added to spiro‐OMeTAD solution for the sake of improving carrier mobility and electrical conductivity of the thin film. The samples were kept in a desiccator for overnight to promote oxidation process of spiro‐OMeTAD. The perovskite device was finally completed by deposition of the metal electrode with 80 nm thick gold coating by thermal evaporator under the high vacuum of 10^−4^ Pa. Alkali metals doped perovskite device was fabricated by adding the certain Na^+^ or K^+^ to perovskite precursor solution. The rest processes were conducted as same as the standard flow.

*Characterizations*: X‐ray diffraction was employed to identify the phase purity of perovskite film (XRD; DX‐2700) via Cu Kα radiation (λ = 1.5416 Å). The absorption spectra of the CH_3_NH_3_PbI_3_ film on compact TiO_2_‐coated FTO were recorded by using an UV–vis spectrometer (Lambda 950, PerkinElmer). The morphology of the perovskite films was investigated on a field emission scanning electron microscopy (SU‐8020, Hitachi). An AFM (MULTIMODE 8, Bruker) was used to image the topography and to measure surface roughness in peak force mode. The thickness of the film was measured by using a Veeco profiler (Dektak 150). The photovoltaic performance was characterized in air without encapsulation under simulated sunlight illumination generated by a solar simulator (XES‐40S2‐CE, San‐Ei Electric, AM 1.5G filter at 100 mW cm^−2^), which was calibrated by using a certified silicon photodiode. *J*–*V* characteristics were obtained by using a source meter (2400, Keithley) at a sweep rate of 0.20 V s^−1^ in forward and backward scan mode. The steady‐state photoluminescence spectra were recorded based on F‐7000 (Shimadzu, Japan) with light source of 150 W halogen tungsten lamp. The TR‐PL spectroscopy was acquired on an Edinburgh Instruments FLS920 fluorescence spectrometer with the time‐correlated single‐photon counting method. The excitation source was a picosecond pulsed diode laser at 406.8 nm with the pulse width 64.2 ps. The IPCE (monochromatic incident photon‐to‐electron conversion efficiency) was performed on the QTest Station 2000ADI system (Crowntech. Inc., USA). The light source was a 150 W halogen tungsten lamp, and the monochromatic light intensity for the IPCE was calibrated by a reference silicon photodiode.

## Conflict of Interest

The authors declare no conflict of interest.

## Supporting information

Supplementary
