# Supplementary material for: Alkali Metal Doping for Improved CH3NH3PbI3 Perovskite Solar Cells
Source: Adv Sci (Weinh). 2017 Dec 21;5(2):1700131. doi: 10.1002/advs.201700131 (PMC5827644; doi:10.1002/advs.201700131)
Supplement: Supplementary file 1 — Supplementary [file ADVS-5-1700131-s001.pdf]

Copyright WILEY-VCH Verlag GmbH & Co. KGaA, 69469 Weinheim, Germany, 2017.

ADVANCED SCIENCE Open Access

Supporting Information

for *Adv. Sci.,* DOI: 10.1002/advs.201700131

Alkali Metal Doping for Improved $CH_3NH_3PbI_3$ Perovskite Solar Cells

Wangen Zhao, Zhun Yao, Fengyang Yu, Dong Yang, and Shengzhong (Frank) Liu**

Alkali Metal Doping for Improved $CH_3NH_3PbI_3$ Perovskite Solar Cells

*Wangen Zhao[a], Zhun Yao[a], Fengyang Yu[a], Dong Yang[a], Shengzhong (Frank) Liu[a,b]**

a. a.Key Laboratory for Applied Surface and Colloid Chemistry, National Ministry of Education; Shaanxi Engineering Lab for Advanced Energy Technology; School of Materials Science and Engineering, Shaanxi Normal University, Xi'an 710062, China.

b. Dalian National Laboratory for Clean Energy, iChEM (Collaborative Innovation Center of Chemistry for Energy Materials); Dalian Institute of Chemical Physics, Chinese Academy of Sciences, Dalian, 116023, China.

Tel & Fax: (180) 9186-3826; Email:szliu@dicp.ac.cn; Liusz@snnu.edu.cn

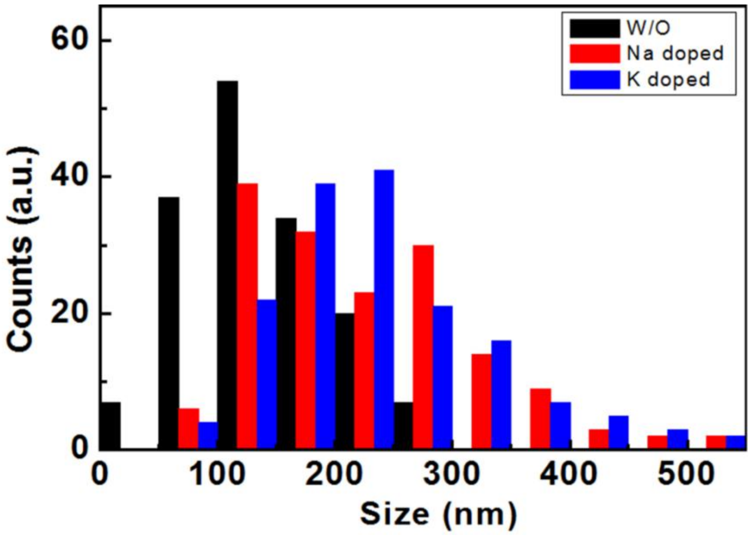

Figure S1. The grain statistical distribution based on the top-view images.

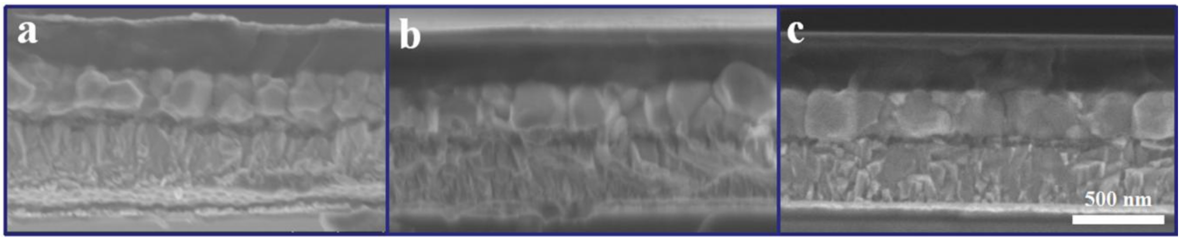

Fig S2. The top-view FE-SEM images of perovskite absorbing layer without (a) and (b) Na and (c) K doping.

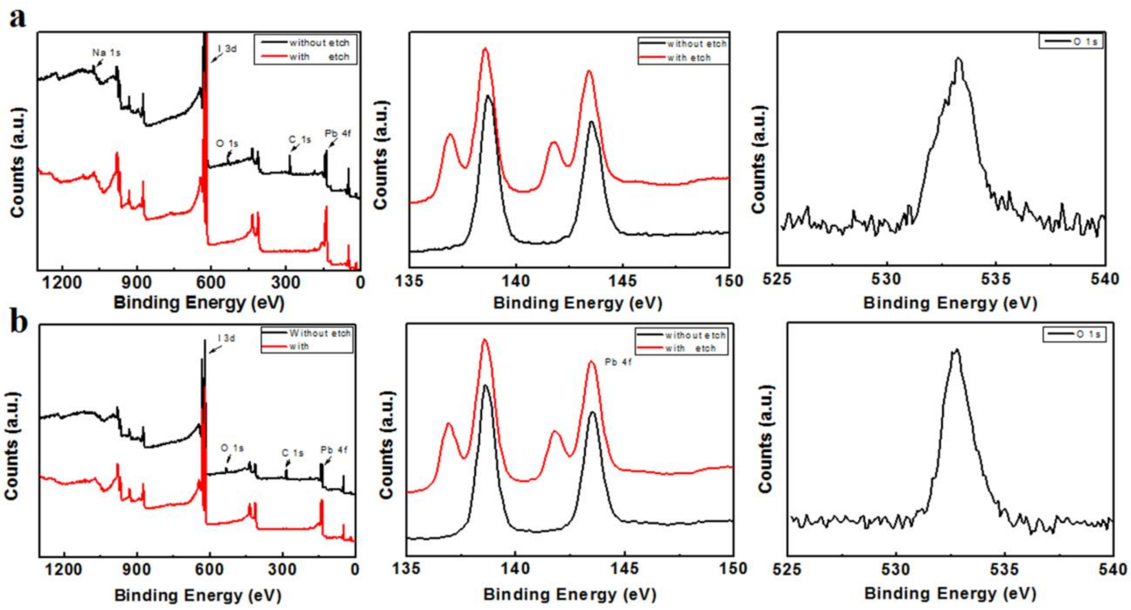

Figure S3. XPS (X-ray photoelectron spectroscopy) of perovskite film with (a) Na^{+} and (b) K^{+} doping. (Note that the black curve were collected by measuring the surface information of sample while red curves were got with Ar^{+} etching).

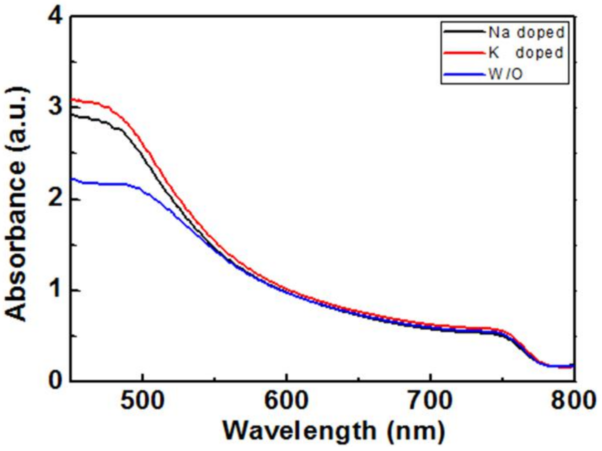

Figure S4. The absorption spectra of perovskite film with different doping cation.

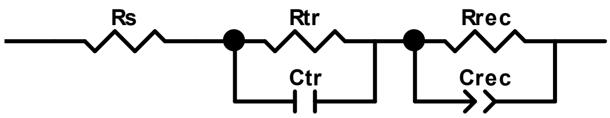

Figure S5. The equivalent circuit model for perovskite solar cells in EIS.

Table S1. EIS parameters of the perovskite cells without and with Na and K doping.

Samples	W/O	Na	K
Rs (Ω)	13.17	11.41	14.78
Rtr (Ω)	18223	15856	29813
Rrec (Ω)	10456	14519	20339
Ctr (F)	3.13×10^{-8}	2.75×10^{-8}	2.55×10^{-8}
Crec (F)	2.27×10^{-7}	2.68×10^{-7}	3.53×10^{-7}

Table S2. The statistical parameters of the $CH_3NH_3PbI_3$ layer with Na^+ doping.

**No.**	***FF***	J_{sc} (mA/cm^2)	V_{oc} (V)	***PCE* (%)**
1	0.78	21.16	1.10	18.16
2	0.75	21.75	1.08	17.61
3	0.76	21.39	1.10	17.88
4	0.78	21.38	1.10	18.34
5	0.76	21.63	1.12	18.41
6	0.76	21.56	1.10	18.02
7	0.78	21.71	1.08	18.29
8	0.77	20.89	1.12	18.12
9	0.76	21.54	1.10	17.94
10	0.78	21.16	1.10	18.16
11	0.80	20.73	1.12	18.57
12	0.77	20.89	1.12	18.12
13	0.78	21.23	1.10	18.22
14	0.76	22.07	1.08	18.11
15	0.78	21.54	1.10	18.48
16	0.77	21.22	1.10	18.07
17	0.77	21.78	1.10	18.44
18	0.76	22.12	1.08	18.16
19	0.76	21.54	1.10	17.94
20	0.77	21.40	1.10	18.13
Average	0.77±0.02	21.43±0.37	1.10±0.02	18.16±0.23

Table S3. The statistical key parameters of the control $CH_3NH_3PbI_3$ layer.

**No.**	***FF***	J_{sc} (mA/cm^2)	V_{oc} (V)	***PCE* (%)**
1	0.70	20.97	1.06	15.56
2	0.71	20.87	1.06	15.71
3	0.72	20.37	1.06	15.55
4	0.70	20.50	1.06	15.21
5	0.74	20.06	1.06	15.68
6	0.74	20.11	1.06	15.77
7	0.72	20.85	1.08	16.21
8	0.74	20.78	1.06	16.30
9	0.68	21.05	1.04	14.89
10	0.70	20.80	1.04	15.14
11	0.75	20.63	1.04	16.09
12	0.74	21.56	1.08	17.23
13	0.73	21.40	1.02	15.93
14	0.74	22.01	1.10	16.10
15	0.72	20.63	1.04	15.45
16	0.73	21.04	1.02	15.67
17	0.70	22.06	1.04	16.06
18	0.70	21.73	1.06	16.12
19	0.71	20.58	1.06	15.49
20	0.72	20.97	1.06	16.00
Average	0.72±0.02	20.95±0.56	1.06±0.02	15.81±0.51

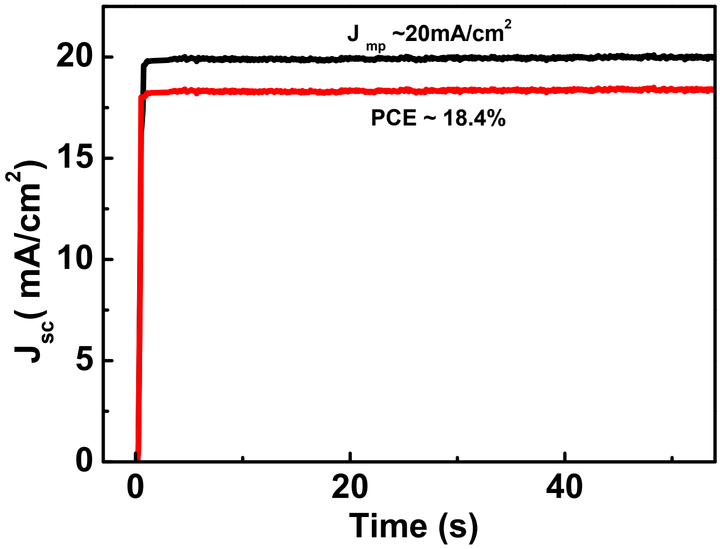

Figure S6. The Photocurrent density and PCE measured as a function of time for the champion device biased at 0.92 V.
